# Prestress Transfer in NSM CFRP-Strengthened RC Structures Under Curing and Service Temperature Effects: Experimental Validation and Analytical Modeling

**DOI:** 10.3390/polym17182492

**Published:** 2025-09-15

**Authors:** Shuang Gong, Peiqi He, Ruogu Wang, Junjin Li, Jun Liu, Miao Su

**Affiliations:** 1 Yunnan Science and Technology Research Institute of Highway, Kunming 650051, China; shuanggong1122@163.com (S.G.); hpq313@163.com (P.H.); jttglcqq@163.com (R.W.); naxiesuiyue@126.com (J.L.); 2 National-Local Joint Laboratory of Engineering Technology for Long-Term Performance Enhancement of Bridges in Southern District, Changsha University of Science & Technology, Changsha 410114, China; 3 School of Civil Engineering, Changsha University of Science and Technology, Changsha 410114, China; junliu97526@163.com

**Keywords:** near-surface-mounted (NSM), carbon fiber-reinforced polymer (CFRP), prestress transmission, adhesive properties, analytical model

## Abstract

This study examines the prestress transmission behavior in near-surface-mounted (NSM) carbon fiber-reinforced polymer (CFRP)-strengthened reinforced concrete structures, with particular emphasis on the effects of temperature. Experimental tests were conducted to evaluate the tensile and shear properties of epoxy adhesives under a range of curing temperatures (20–100 °C) and ambient service temperatures (0–80 °C). The results reveal an inverse exponential relationship between curing time and temperature. Notably, adhesive strength declines significantly above 60 °C and the adhesive loses functionality at 80 °C. Building on these findings, an analytical model was developed to predict prestress transfer length, CFRP strain distribution, and interfacial shear stress. The model incorporates effective bond stiffness and a prestress reduction coefficient to account for varying prestress levels (10–50%). Parametric analyses identify the CFRP elastic modulus, cross-sectional geometry, adhesive thickness, and degree of curing as critical factors influencing prestress transmission. The model’s predictions were validated against experimental data, demonstrating its reliability. Overall, this work provides a theoretical foundation for optimizing the design of NSM CFRP-strengthened structures under complex thermal conditions.

## 1. Introduction

Carbon fiber-reinforced polymer (CFRP) has become a pivotal material in civil engineering, particularly for strengthening reinforced concrete (RC) structures [1,2,3,4,5]. Its widespread adoption is primarily attributed to its high strength-to-weight ratio, excellent corrosion resistance, and superior installation efficiency compared to traditional steel reinforcement. CFRP strengthening systems effectively restore and upgrade aging RC structures by enhancing load-bearing capacity and extending service life. However, despite these advantages, practical implementation continues to face significant challenges, especially under complex environmental conditions. Among these, temperature fluctuations are especially critical [6,7,8], as they directly affect the interfacial bonding between CFRP and concrete—a factor fundamental to the reliability of strengthening systems.

Near-surface-mounted (NSM) technology has attracted considerable attention due to its distinctive strengthening mechanism [9,10,11]. In this approach, the strengthening material is embedded within the concrete cover, achieving three- or four-sided bonding, which significantly enhances bond performance and makes NSM technology particularly suitable for slabs or bars with a low height-to-width ratio. Encapsulating the strengthening material within the concrete cover also improves stability and durability. Furthermore, the application of prestress in NSM CFRP-strengthened structures further increases flexural and shear capacities, durability, and overall load-bearing performance [12,13,14]. Effective prestress transfer is essential to ensure synergistic interaction between CFRP and concrete, thereby maximizing strengthening efficiency. Prestress is transferred from the CFRP ends into the concrete, a process fundamentally governed by adhesive performance. The efficiency of prestress transfer directly influences stress distribution in the CFRP and, consequently, the overall strengthening effect. This issue is particularly significant in gradually anchored prestressed beams [15,16,17], where the prestress level decreases at the CFRP ends and adhesive curing efficiency is enhanced by thermal curing. In such cases, a comprehensive understanding of the prestress transfer mechanism is crucial.

Although extensive research has focused on the overall performance of CFRP-strengthened structures, detailed investigations into the prestress transfer mechanisms in NSM CFRP systems remain limited. Most existing studies primarily address the intrinsic mechanical properties of CFRP, adhesive characteristics, and the global mechanical behavior of strengthened structures [18,19,20], while systematic examinations of the mechanisms governing prestress transfer at the CFRP–concrete interface are scarce. Furthermore, comprehensive theoretical and experimental research on the effects of adhesive performance—under varying curing and ambient temperatures—on prestress transfer, as well as accurate modeling of CFRP strain distribution after prestress release, is lacking. These gaps hinder the precise prediction and evaluation of NSM CFRP-strengthened structures under real-world conditions and limit the further optimization and broader application of NSM CFRP technology. Recent research by Jafari et al. [21] n post-heated steel-fiber SCC beams strengthened with NSM-CFRP strips demonstrates that elevated temperatures degrade bond and flexural capacity. Their findings highlight the necessity of quantifying thermally induced adhesive softening and its impact on prestress transfer, which is the specific focus of this study.

To address these limitations, this study investigates the prestress transfer characteristics of NSM CFRP-strengthened structures. First, adhesive performance tests are conducted to evaluate variations in tensile and shear properties under different curing and ambient temperatures. Second, based on theoretical mechanics, a micro-element at the CFRP end is selected, and the effective bond stiffness is defined by integrating key parameters such as the modulus and stiffness of both the adhesive and the concrete. By incorporating the prestress level and geometric failure assumptions, the strain distribution in CFRP after prestress release is mathematically derived. The accuracy of the derived formula is then validated against experimental data, followed by a parametric analysis of critical variables to clarify the influence of various factors on prestress transfer. This study aims to establish a rigorous and precise theoretical foundation for the design, construction, and performance assessment of NSM CFRP-strengthened structures, thereby promoting the broader application and further development of NSM CFRP technology in engineering practice.

## 2. Experimental Program

### 2.1. Test Series and Materials Properties

The mechanical properties of the epoxy binder were assessed through two primary tests: (1) a tensile performance test, which measured the tensile strength of the cured resin and evaluated the effects of different curing conditions; and (2) a shear performance test, which determined the shear strength of the resin under various curing regimes to identify optimal curing durations at specific temperatures.

Three main materials were used in the experiment: CFRP strips, epoxy resin adhesive, and concrete. The CFRP strips (ASTEC CT124-2, Dextra Building Products (Guangzhou, China)), with a cross-sectional area of 16 mm × 2 mm, were employed for NSM bond strengthening. According to ASTM D3039 [22], these strips exhibited an average tensile strength of 2564.3 MPa, an elastic modulus of 140.7 GPa, and an ultimate strain of 1.96%. The adhesive used was Sikadur-30 CN, a two-component epoxy resin, which, as specified by GB50728-2011 [23], had an average tensile modulus of 3.2 GPa and a tensile strength of 40 MPa. The concrete, supplied by Hunan Ruixin Company (Changsha, China), was commercial-grade with a designed compressive strength of C40. The material properties used in the performance tests are summarized in Table 1.

### 2.2. Specimen Design and Preparation

**Figure 1:**
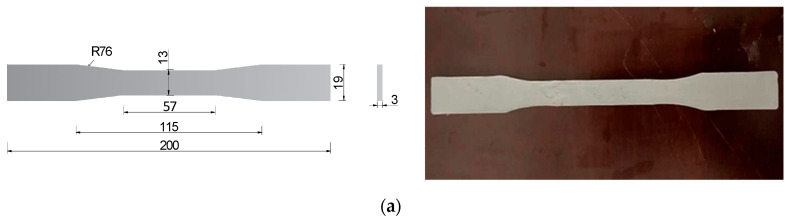

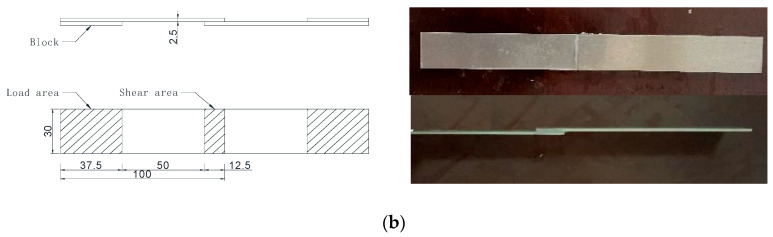
Geometry and configuration of specimens for mechanical testing (unit: mm). (Reproduced with permission from [26]. Copyright 2025, Acta Materiae Compositae Sinica). (**a**) Tensile specimen; (**b**) shear specimen.

Figure 1 presents the geometry and configuration of the specimens used for the mechanical property tests of the epoxy resin binder. The tensile property test, conducted in accordance with ASTM D638-10 [24], utilized dog-bone-shaped specimens (Figure 1a). The shear property test followed the Chinese national standard GB/T 7124-2008 [25], and used specimens formed by bonding two aluminum sheets (Figure 1b). To ensure concentric loading and uniform stress distribution, uniform-thickness gaskets were placed at both ends of each specimen.

### 2.3. Test Setup, Instrumentation and Procedures

**Figure 2:**
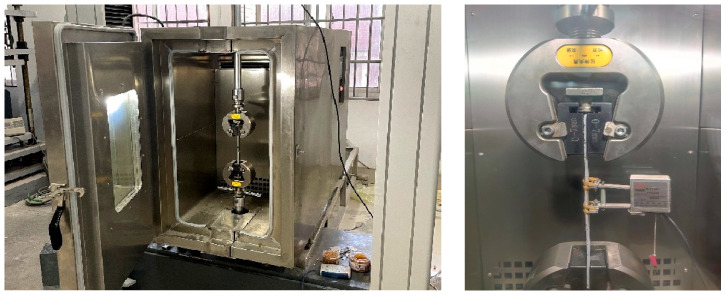
MTS Landmark electro-hydraulic servo testing system for quasi-static tensile testing. (Reproduced with permission from [26]. Copyright 2025, Acta Materiae Compositae Sinica).

Quasi-static tensile tests were carried out using an MTS Landmark-370.25 (MTS Systems, Eden Prairie, MN, USA) electro-hydraulic servo testing system equipped with an MTS high/low-temperature chamber, as shown in Figure 2. Pneumatic fixtures were employed for clamping, with the lower chuck fixed and the upper chuck moving vertically. The temperature chamber provided a control range from −100 to 300 °C and maintained a temperature accuracy of ±0.5 °C. Displacement-controlled loading was adopted, with a tensile test rate of 2 mm/min and a shear test rate of 0.3 mm/min. The upper end of the specimen was first clamped, and the ambient temperature was set prior to testing. Once the chamber temperature reached approximately 3 °C below the target value, the chamber was opened to clamp the lower end of the specimen, then closed to resume heating. After reaching the specified temperature, the chamber was maintained at that temperature for 15 min before testing commenced. Throughout the test, temperature, load, and displacement were continuously recorded by sensors integrated into the loading system. The maximum load at each temperature was recorded as the failure load, and the cross-sectional dimensions of the failed specimen were measured using an electronic vernier caliper.

To directly validate the transfer mechanisms in actual NSM configurations, supplementary pull-out and beam-end tests on CFRP–concrete specimens were conducted at temperatures ranging from 0 to 80 °C. The results, as reported in [26], confirm the analytical predictions presented in this study.

## 3. Results

### 3.1. Effect of Curing Temperature on Tensile Strength and Elasticity

**Figure 3:**
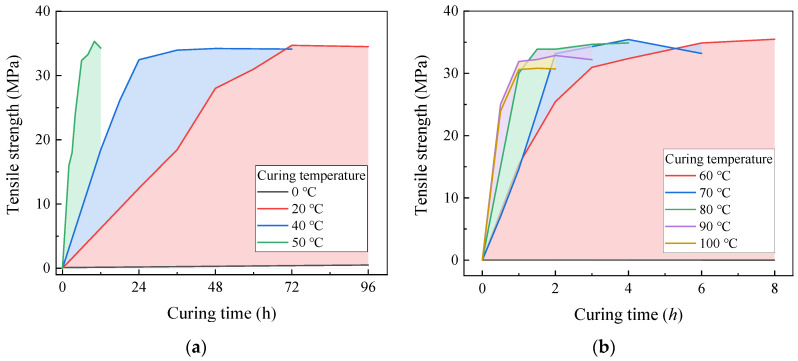
Effect of curing temperature on tensile strength of epoxy resin. (Reproduced with permission from [26]. Copyright 2025, Acta Materiae Compositae Sinica). (**a**) Tensile strength at 0–50 °C; (**b**) tensile strength at 60–100 °C.

The tensile and shear properties of the epoxy resin binder, tested as described in Section 2.2, are summarized in Figure 3. The results indicate that the tensile strength of the epoxy resin generally increases with curing time, reaching a maximum value of approximately 35 MPa for all specimens. However, when the curing temperature exceeds 80 °C, the tensile strength decreases by up to 10%. This reduction is accompanied by yellowing and the formation of dense bubbles, which result from the accelerated reaction rate at excessively high curing temperatures. Such conditions induce rapid heat generation, bubble formation, and internal stress development, ultimately compromising the tensile properties of the epoxy resin.

**Figure 4:**
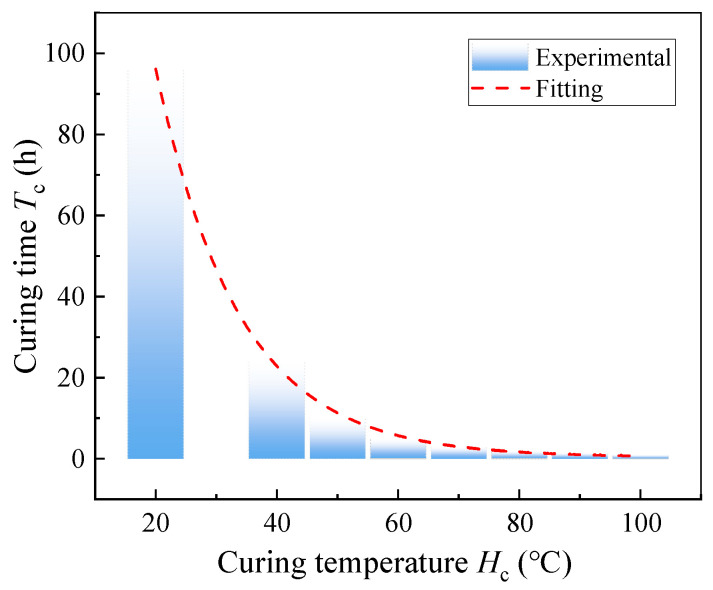
Relationship between curing time and curing temperature. (Reproduced with permission from [26]. Copyright 2025, Acta Materiae Compositae Sinica).

Specimens cured at 20 °C for 96 h are considered fully cured and serve as the benchmark for comparison. As depicted in Figure 4, the relationship between curing time and temperature within the studied range (20–100 °C) is described by Equation (1):

(1) $$T_{c}=0.49+409.18\times 0.93^{H_{C}},$$

where *H*_c_ represents the environmental temperature during curing (°C) and *T*_c_ denotes the required curing time (*h*) to achieve the specified strength.

### 3.2. Effect of Test Temperature on Tensile Properties

**Figure 5:**
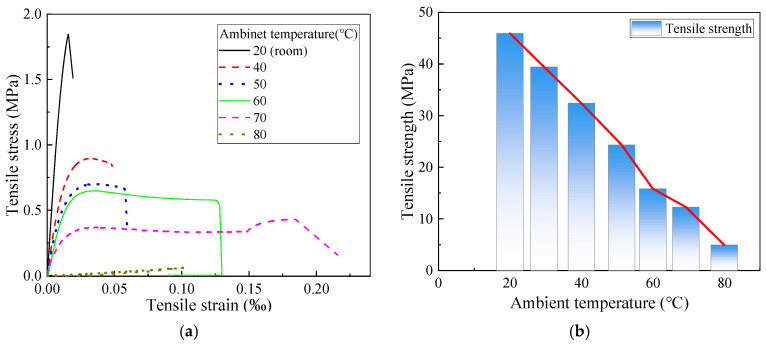
Tensile behavior of epoxy resin at different ambient temperatures. (Reproduced with permission from [26]. Copyright 2025, Acta Materiae Compositae Sinica). (**a**) Stress–strain curve; (**b**) tensile strength-test ambient temperature curve.

**Figure 6:**
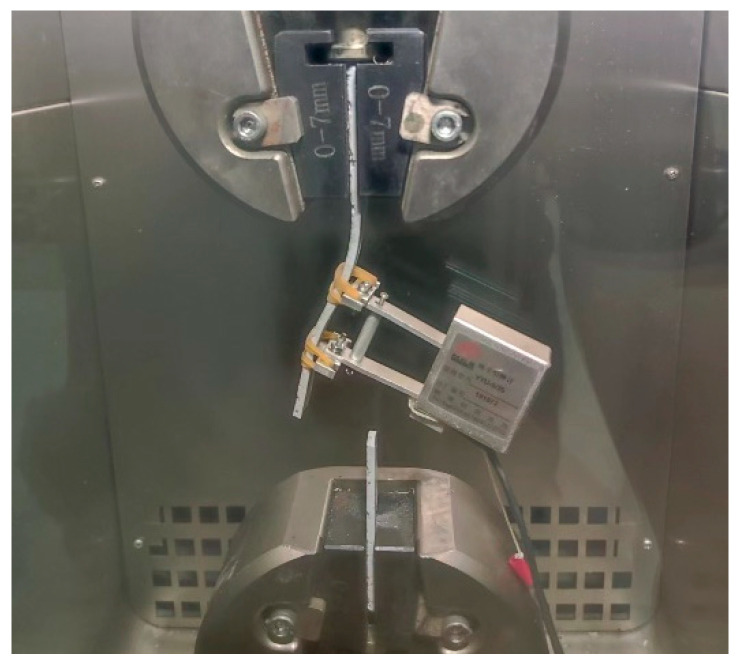
Tensile failure mode of epoxy resin at 60 °C ambient temperature. (Reproduced with permission from [26]. Copyright 2025, Acta Materiae Compositae Sinica).

Figure 5 presents the stress–strain curves and tensile strength of epoxy resin specimens cured at 20 °C for 96 h and tested under various ambient temperatures. The results indicate that tensile strength decreases as the test temperature increases. At 40 °C, the tensile strength is reduced to 70% of its value at 20 °C, and the stress–strain curve exhibits elastoplastic behavior. When the test temperature reaches 60 °C, the tensile strength further declines to 30% of its original value, as the temperature approaches the glass transition temperature (*T*_g_). This proximity leads to significant softening and pronounced plastic deformation of the epoxy resin. The failure mode observed at 60 °C is shown in Figure 6. At 80 °C, the epoxy resin exhibits negligible tensile strength. With the increase in temperature, irreversible segmental sliding and dislocation gradually occur inside the epoxy resin, leading to a gradual increase in residual deformation, thus resulting in the manifestation of elastoplastic behavior.

### 3.3. Effect of Test Ambient Temperature on Shear Properties

**Figure 7:**
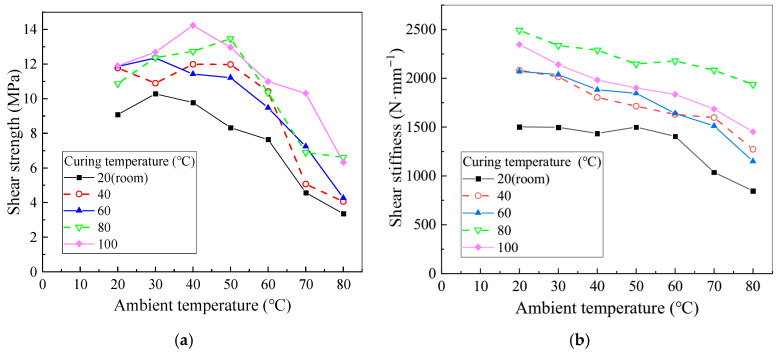
Shear performance at different curing and ambient temperatures. (Reproduced with permission from [26]. Copyright 2025, Acta Materiae Compositae Sinica). (**a**) Shear strength test ambient temperature; (**b**) shear stiffness test ambient temperature.

Following the tensile property tests, the shear properties of the epoxy resin were systematically evaluated under various curing and test ambient temperatures. The results, shown in Figure 7, provide insights into both shear strength and stiffness. Shear strength is defined as the ultimate load divided by the bonded area, while shear stiffness is determined as the initial slope of the load–displacement curve.

As illustrated in Figure 7a, specimens cured at 20 °C exhibit significantly lower shear strength compared to those cured at higher temperatures. Specimens cured at temperatures between 40 °C and 100 °C demonstrate notable improvements in shear strength, highlighting the adverse effect of low curing temperatures on the curing reaction and, consequently, on epoxy performance. In contrast, elevated curing temperatures enhance the curing reaction and improve mechanical performance within an optimal range. However, further increases in curing temperature beyond this range result in a gradual decrease in shear strength due to bubble formation and the development of internal stresses. Initially, specimens cured at higher temperatures display a more pronounced increase in shear strength, followed by a slower decline as the curing temperature continues to rise.

Figure 7b shows that shear stiffness generally decreases with increasing ambient temperature. However, for specimens cured at 20 °C, an anomalous increase in stiffness is observed at 50 °C. This phenomenon is attributed to incomplete curing during the initial reaction, which continues as the temperature rises, although the post-curing effect is limited. For specimens cured within the range of 20 °C to 80 °C, higher curing temperatures generally result in increased shear stiffness, although the difference in shear stiffness between specimens cured at 40 °C and 60 °C is minimal. Notably, specimens cured at 100 °C exhibit lower shear stiffness than those cured at 80 °C, likely due to the excessive curing temperature reduces the integrity of the colloid cross-linking network and causes bubble formation, which reduces the effective bonding area and thereby diminishes shear performance, which is consistent with the phenomena observed by Esposito and Firmo [27,28] in their experiments.

## 4. Prestress Transmission Behavior

This section presents an analytical approach for predicting the distribution of prestress transfer length, tensile strain, and shear stress along CFRP strips, explicitly considering the effects of the adhesive’s elastic modulus and thickness. The proposed approach is validated against experimental results, followed by a parametric study and the derivation of a predictive formula.

### 4.1. Analytical Approach

The analytical model is developed under the following assumptions:

(1) All materials behave in a linear elastic and isotropic manner, the strength of CFRP is only considered along the fiber direction;
(2) Perfect bonding exists between the steel bars, CFRP, and surrounding concrete, with no relative slip;
(3) The strain distribution in the concrete, longitudinal steel bars, and CFRP conforms to the plane section assumption.

After prestress release, the RC beam achieves equilibrium. Considering an infinitesimal segment *dx*, the application of static equilibrium yields Equation (2), which relates the interfacial bond shear stress along *dx* to the tensile strain and thickness of the CFRP:

(2) $$\tau =\frac{a_{f}}{2}\cdot \frac{d\sigma_{f}}{dx},$$

where *τ* represents the interfacial shear stress, *dσ*_f_ denotes the tensile strain of the CFRP, and *a*_f_ refers to the thickness of the CFRP strip.

**Figure 8:**
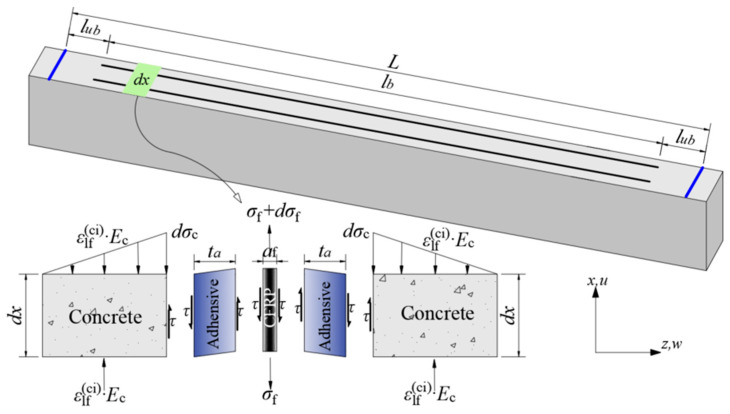
Interface stress distribution per unit length.

**Figure 9:**
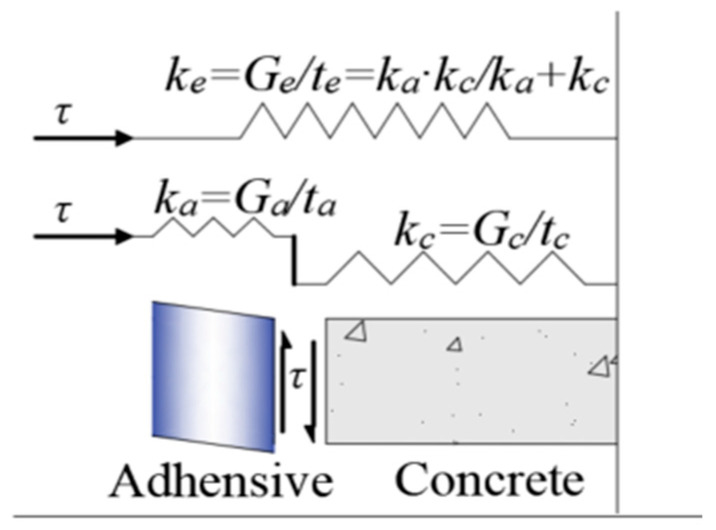
Schematic diagram of equivalent spring element at bonding interface.

As illustrated in Figure 8, the tensile stress in the CFRP strip is primarily transferred to the concrete surface via shear stress developed within the adhesive bonding layer. Assuming linear elastic behavior of the adhesive and no relative slip at the bonding interface, the tensile stress in the CFRP is balanced by the shear stresses in both the adhesive and the groove wall concrete. In NSM strengthening, the combined bonding stiffness of the adhesive and the groove wall concrete surrounding the CFRP strip is defined as the effective bonding stiffness, *k*_e_ [29], as shown in Figure 9, and is calculated using Equation (3):

(3) $$k_{e}=\frac{G_{e}}{t_{e}}=\frac{k_{a}\cdot k_{c}}{k_{a}+k_{c}},$$

where *k*_a_ = *G*_a_/*t*_a_ is the shear stiffness of the adhesive (*G*_a_ and *t*_a_ represent the shear modulus and thickness of the adhesive layer, respectively), and *k*_c_ = *G*_c_/*t*_c_ is the shear stiffness of the groove wall concrete (*G*_c_ and *t*_c_ denote the shear modulus and thickness of the concrete cover, respectively).

Assuming both concrete and adhesive are isotropic, their shear moduli can be determined by Equation (4):

(4) $$G_{c}=\frac{E_{c}}{2(1+\upsilon_{c})},G_{a}=\frac{E_{a}}{2(1+\upsilon_{a})},$$

where *v*_c_ and *v*_a_ are the Poisson’s ratios of concrete and adhesive, respectively.

**Figure 10:**
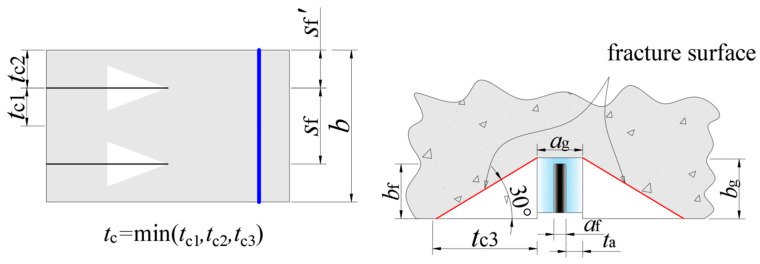
Layout of CFRP and schematic of interface failure.

The thickness of the concrete cover, *t*_c_, at failure depends on the strengthening method. For externally bonded (EB) strengthening, *t*_c_ is typically 2.5–3 times the maximum aggregate diameter, or 40–50 mm [28]. For NSM-strengthened beams, *t*_c_ is calculated using Equation (5):

(5) $$t_{c}=\min(t_{c1},t_{c2},t_{c3})=\min\left({s_{f}}^{\prime },\frac{s_{f}}{2},\frac{b_{g}}{\tan(30^{\circ })}\right),$$

where *s*_f_ denotes the spacing between adjacent CFRP strips, and *s*_f_’ represents the distance from the outermost CFRP strip to the beam side. According to Lorenzis [10] and as illustrated in Figure 10, the fracture propagation path for NSM CFRP forms a 30° angle with the tensile concrete surface. Based on the previous study [30], the length of the opposite side of the fracture path is adjusted to match the groove depth.

Given the linear elastic behavior of all materials, the bond shear stress can be expressed by Equation (6):

(6) $$\tau =G_{e}\cdot \gamma \to \gamma =\frac{du}{dz}+\frac{dw}{dx},$$

where *γ* represents the shear strain, and *u* and *w* denote the longitudinal and transverse displacements of the bond layer, respectively.

By combining Equations (2) and (6) and differentiating with respect to *x*, Equation (7) is derived:

(7) $$\frac{d^{2}\sigma_{f}}{dx^{2}}=\frac{2G_{e}}{a_{f}}\left(\frac{d^{2}u}{dx\cdot dz}+\frac{d^{2}w}{dx^{2}}\right).$$

Here, the second-order differential of the lateral displacement can be neglected due to symmetry. The derivatives of the longitudinal displacement with respect to the longitudinal and lateral directions can then be reformulated as Equation (8):

(8) $$\frac{d^{2}u}{dxdz}=\frac{d}{dz}\left(\frac{du}{dx}\right)=\frac{d}{dz}\left(\frac{du_{c}}{dx}-\frac{du_{f}}{dx}\right)=\frac{1}{t_{e}}\left(\varepsilon_{c}-\varepsilon_{\mathrm{lsf}}\right),$$

where *u*_c_ and *u*_f_ denote the longitudinal displacements of the concrete and CFRP within the cross-section, respectively; *ε*_c_ represents the compressive strain of the concrete in the same cross-section; and *ε*_lsf_ refers to the reduction in tensile strain in the CFRP caused by prestress release. This strain is calculated as *ε*_lsf_ = *ε*_fp_ − *ε*_f_, where *ε*_fp_ is the design value of the prestress strain and *ε*_f_ is the actual tensile strain in the CFRP.

Upon prestress release, compressive strain develops in the concrete section of the micro-element due to the negative bending moment. According to the plane section assumption, this compressive strain equals the short-term strain loss in the CFRP (*ε*_c_ = *ε*_lf_). The short-term strain loss in the prestressing tendon is defined as *ε*_ef_ = *ε*_fp_ − *ε*_lf_. Substituting these relationships, along with Equations (3) and (8), into Equation (7) yields Equation (9):

(9) $$\frac{d^{2}\sigma_{f}}{dx^{2}}-\frac{2G_{e}}{t_{e}\cdot a_{f}\cdot E_{f}}\cdot \sigma_{f}=-\frac{2G_{e}}{t_{e}\cdot a_{f}}\left(\varepsilon_{\mathrm{fp}}-\varepsilon_{\mathrm{lf}}\right)\to \frac{d^{2}\sigma_{f}}{dx^{2}}-\frac{2k_{e}}{a_{f}\cdot E_{f}}\cdot \sigma_{f}=-\frac{2k_{e}}{a_{f}}\left(\varepsilon_{\mathrm{ef}}^{\left(ci\right)}\right).$$

This equation can be reformulated as

(10) $$\frac{d^{2}\sigma_{f}}{dx^{2}}-B^{2}\cdot \sigma_{f}=-B^{2}\cdot E_{f}\cdot \varepsilon_{\mathrm{ef}}^{\left(ci\right)}\to B^{2}=\frac{2k_{e}}{a_{f}\cdot E_{f}},$$

where *B* denotes the stiffness coefficient.

The general solution to Equation (10) is given by

(11) $$\sigma_{f}(x)=C_{1}e^{Bx}+C_{2}e^{-Bx}+E_{f}\cdot \varepsilon_{\mathrm{ef}}^{\left(ci\right)}.$$

Consequently, the shear stress can be represented by Equation (12):

(12) $$\tau_{\left(x\right)}=\frac{a_{f}}{2}\left(B\cdot C_{1}e^{Bx}-B\cdot C_{2}e^{-Bx}\right).$$

Applying the boundary conditions (*x* = 0, *σ*_f_ = 0; *x* = *L*_b_/2, *τ* = 0), the constants *C*_1_ and *C*_2_ can be determined as presented in Equation (13):

(13) $$C_{1}=\frac{-E_{f}\cdot \varepsilon_{\mathrm{ef}}^{\left(ci\right)}}{\left(1+e^{BL_{b}}\right)},C_{2}=\frac{-E_{f}\cdot \varepsilon_{\mathrm{ef}}^{\left(ci\right)}\cdot e^{BL_{b}}}{\left(1+e^{BL_{b}}\right)},$$

where *e^BLb^* is significantly larger than both *B* and *L*_b_, the contribution of *C*_1_ becomes negligible and *C*_2_ can be reformulated accordingly. Thus, Equations (11) and (12) are revised as follows:

(14) $$\sigma_{f}(x)=E_{f}\cdot \varepsilon_{\mathrm{ef}}^{\left(ci\right)}(1-e^{-Bx}),$$

(15) $$\tau (x)=\frac{a_{f}\cdot E_{f}\cdot B\cdot \varepsilon_{\mathrm{ef}}^{\left(ci\right)}\cdot e^{-Bx}}{2}.$$

### 4.2. Prestress Transmission Length

After the adhesive has fully cured, the tensioning devices at both ends of the CFRP are removed, introducing prestress at the CFRP end, which gradually increases toward the mid-span. The region where the prestress transitions from zero to the design value is defined as the prestress transfer length (*TL*_a_). The variation in prestress within this region induces shear stress at the CFRP–adhesive–concrete bonding interface.

**Figure 11:**
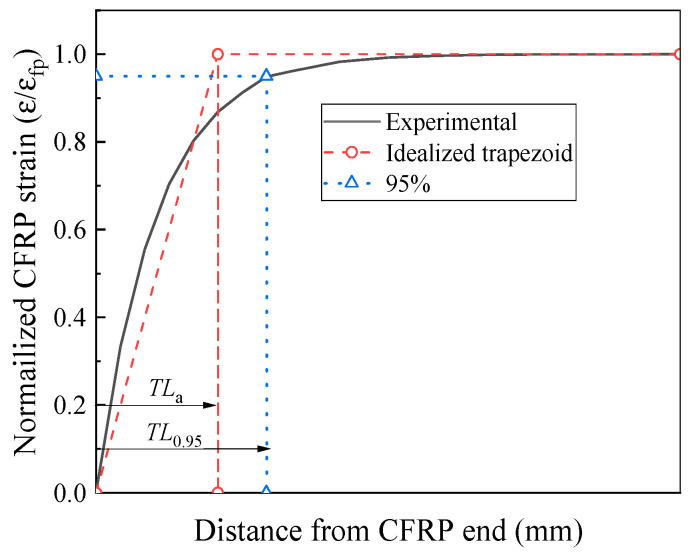
Prestress transmission length of CFRP.

In this study, the equal area method is employed to calculate the prestress transfer length *TL*_a_, as illustrated in Figure 11. The vertical axis of the strain distribution diagram is normalized by the design strain value *ε*_fp_. The strain variation is idealized as a trapezoid of unit height, such that its area equals that enclosed by the actual strain distribution curve, as expressed in Equation (16):

(16) $$\int_{0}^{L_{b}/2}\frac{\sigma_{f}(x)}{E_{f}\cdot \varepsilon_{\mathrm{fp}}}d(x)=\left(\frac{\left(L_{b}/2)+((L_{b}/2)-TL_{a}\right)}{2}\right)\cdot \left(\frac{\varepsilon_{\mathrm{ef}}^{\left(ci\right)}}{\varepsilon_{\mathrm{fp}}}\right).$$

Substituting Equation (14) into (16) yields Equation (17):

(17) $$\int_{0}^{L_{b}/2}\frac{E_{f}\cdot \varepsilon_{\mathrm{ef}}^{\left(\mathrm{ci}\right)}\left(1-e^{-Bx}\right)}{E_{f}\cdot \varepsilon_{\mathrm{fp}}}d(x)=\left(\frac{\varepsilon_{\mathrm{ef}}^{\left(\mathrm{ci}\right)}}{\varepsilon_{\mathrm{fp}}}\right)\cdot \left(x+\frac{e^{-Bx}}{B}\right)=\left(\frac{L_{b}-TL_{a}}{2}\right)\cdot \left(\frac{\varepsilon_{\mathrm{ef}}^{\left(\mathrm{ci}\right)}}{\varepsilon_{\mathrm{fp}}}\right).$$

Accordingly, the prestress transfer length *TL*_a_ can be expressed by Equation (18):

(18) $$TL_{a}=\frac{2(1-e^{-BL_{b}/2})}{B},$$

where the term *e*^*BLb*/2^ is significantly smaller than *B* and *L*_b_ and can be neglected, leading to the simplified form in Equation (19).

(19) $$TL_{a}=\frac{2}{B}\to B={\left(\frac{2k_{e}}{a_{f}\cdot E_{f}}\right)}^{0.5}.$$

As indicated by Equation (19), the prestress transfer length is closely related to the stiffness parameters of the interface surrounding the NSM CFRP.

### 4.3. Prediction Results

**Figure 12:**
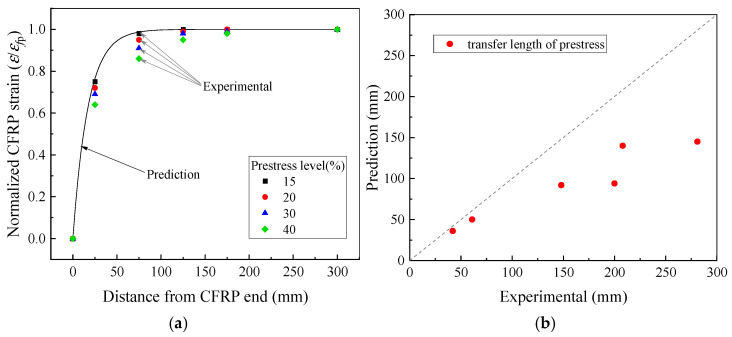
Prediction of prestress transfer length. (**a**) Distribution of CFRP strain; (**b**) transfer length of prestress.

As shown in Figure 12, Rezazadeh [31,32] applied this approach to calculate the CFRP strain distribution and the prestress transfer length after prestress tensioning in experimental studies. The method accurately predicted the CFRP strain distribution for specimens with a 15% prestress level but underestimated the prestress transfer length for prestress levels of 20%, 30%, and 40%.

**Figure 13:**
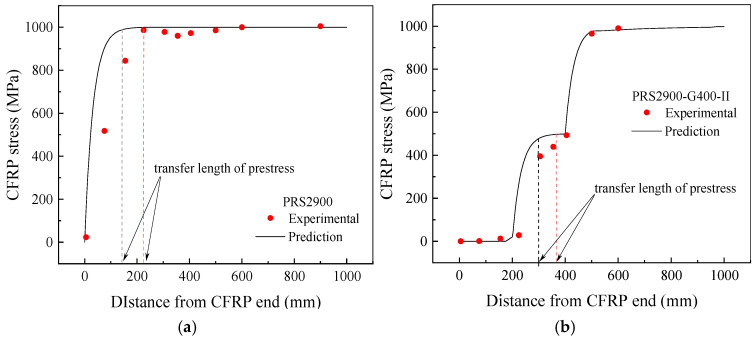
Distribution of CFRP strain in prestressed specimens. (**a**) Specimen PRS2900; (**b**) Specimen PRS2900-G400-II.

Figure 13 presents the CFRP strain distribution in the shear span of both the conventional prestressed strengthened beam PRS2900 and the gradually anchored prestressed strengthened beam [15]. The results indicate that the predictive model overestimates the stress transfer efficiency at the CFRP–concrete interface, resulting in a calculated prestress transfer length that is significantly smaller than the actual value. This discrepancy arises because the CFRP strain was normalized during calculation, without considering the influence of the prestress level on the stiffness of the bonding interface. Higher prestress levels require a longer bonding length to achieve effective stress transfer.

**Figure 14:**
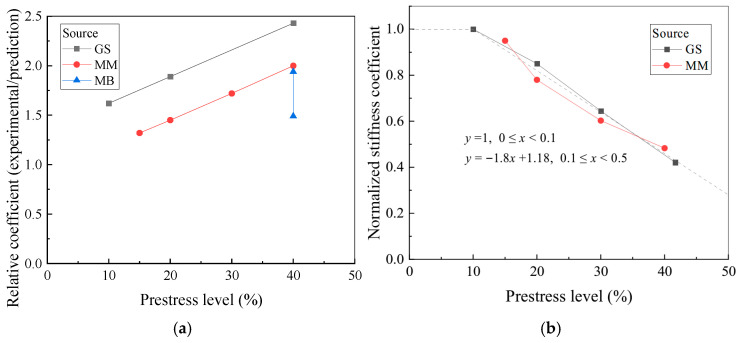
Influence of prestress level on CFRP–concrete interface behavior. (**a**) Relative coefficient; (**b**) normalized stiffness coefficient.

In calculating the prestressed length, the stiffness coefficient *B* is a critical parameter, defined as the ratio of the effective stiffness *k*_e_ of the bonding interface to the product of the width *a*_f_ and elastic modulus *E*_f_ of the strengthening material. The effective stiffness of the bonding interface is determined by the shear stiffnesses *k*_c_ and *k*_a_ of the concrete and adhesive, respectively. After determining the stiffness coefficient *B*, a prestress transfer coefficient *α*_p_ is introduced to account for the effect of the prestress level. Analysis of Rezazadeh’s test beam [20] and previously tested strengthened beams [14,15], as shown in Figure 14a, demonstrates a direct proportional relationship between the stress transfer level at the CFRP–concrete interface and the prestress level in the CFRP. Figure 14 presents the reduction in the stiffness coefficient at different prestress levels, using the initially calculated value as a reference. When the prestress level is below 10%, Equation (18) accurately simulates the interfacial stress transfer behavior. However, when the prestress level does not exceed 50%, the stiffness coefficient must be adjusted. The reduction coefficient *α*_p_ is calculated using Equation (20):

(20) $$\left\{\begin{array}{l} \alpha_{p}=1,0\leq p\%<0.1\\ \alpha_{p}=-1.8p\%+1.18,0.1\leq p\%<0.5 \end{array}\right.,$$

where *α*_p_ represents the influence factor of the prestress level on the stiffness coefficient *B*, with 0 < *α*_p_ ≤ 1; *p*% denotes the ratio of the applied prestress level to the material’s ultimate strength.

**Figure 15:**
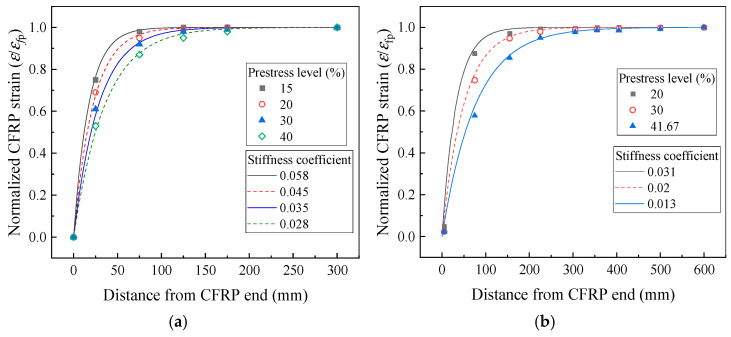
Distribution of CFRP strain from experimental studies. (**a**) Experiments of Rezazadeh; (**b**) Experiments of Gong.

The stiffness coefficient B is adjusted based on the prestress level using Equation (20). As shown by the computational results in Figure 15, the revised formula accurately predicts both the stress transfer behavior and the CFRP strain distribution across various prestress levels, enabling a more precise determination of the shear stress induced at the CFRP end due to prestress transfer. It should be noted that multiple parameters—including the elastic modulus and dimensions of CFRP, the degree of adhesive curing, groove size, and surface treatment method of CFRP—can influence interfacial stress transfer. Further investigation is required to quantitatively characterize the effects of these parameters.

**Figure 16:**
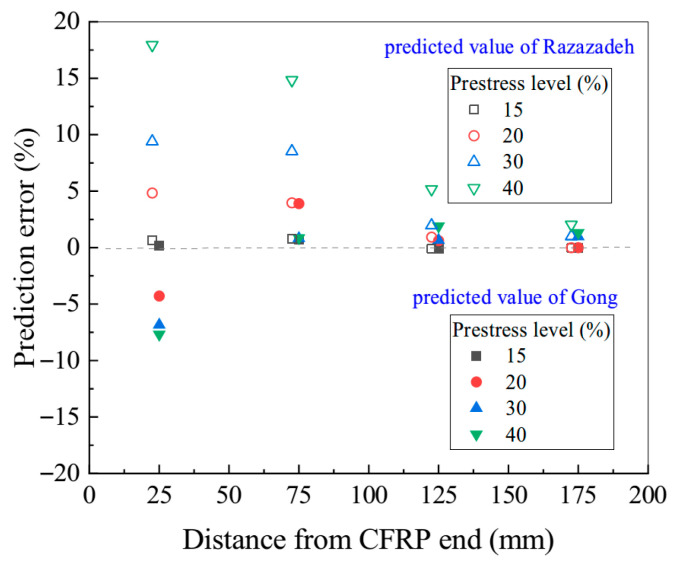
Effect of prestress level on prediction accuracy.

Figure 16 demonstrates that, when applied to Rezazadeh’s experimental data [31,32], the proposed method with the stiffness coefficient correction restricts the prediction error of the prestress transfer length to within 10% for all test scenarios. This result represents a significant improvement over the original model, which exhibited a maximum error of up to 20%. The largest prediction error occurs near the CFRP end, where contraction is most pronounced. As the measurement point moves away from the end, the degree of contraction decreases, and the prediction error rapidly diminishes. Moreover, higher prestress levels result in greater contraction amplitudes and steeper strain gradients between adjacent measurement points, leading to increased prediction discrepancies.

### 4.4. Parametric Analysis

A simplified parametric analysis was conducted based on the established analytical approach. The parameters investigated included the elastic modulus of concrete, CFRP, and adhesive; the thicknesses of the CFRP and adhesive layers; and the curing condition of the adhesive. To highlight the effects of these factors on the CFRP strain distribution, shear stress distribution, and prestress transfer length, the computational results were normalized by the initial CFRP strain, the maximum shear stress at the bonded end, and the prestress transfer length, respectively. Comparative analysis was performed using specimens with variation coefficients of 0.5, 1, and 2. It is important to note that this method tends to overestimate the shear stress within the 20 mm segment near the CFRP end; therefore, data from this region should be excluded during result interpretation.

(1) Material properties

**Figure 17:**
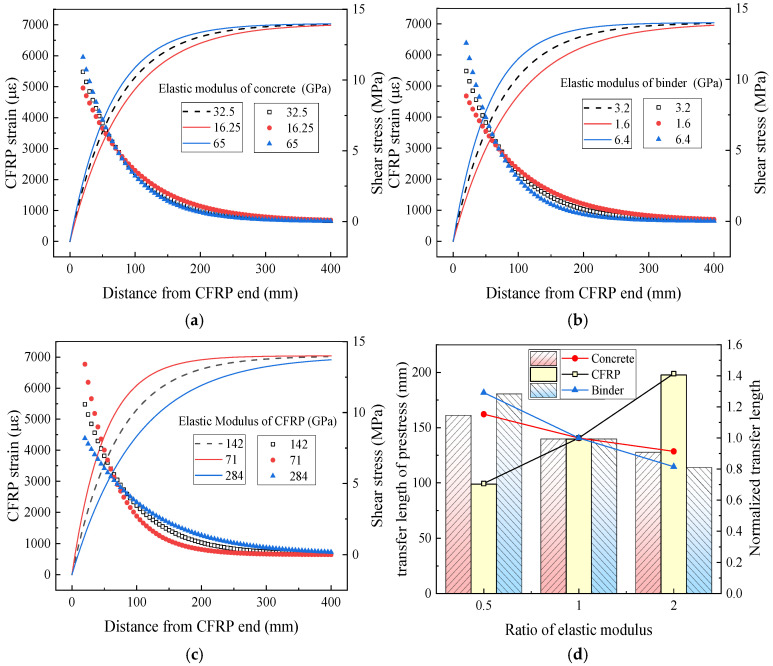
Influence of elastic modulus on prestress transfer behavior. (**a**) Elastic modulus of concrete; (**b**) elastic modulus of binder; (**c**) elastic modulus of CFRP; (**d**) transfer length of prestress.

As illustrated in Figure 17, the elastic modulus of each constituent material influences the strain distribution in the CFRP, with the modulus of concrete exerting the least effect and that of CFRP the most pronounced. An increase in the elastic modulus of either concrete or adhesive results in a reduction in prestress transfer length. Specifically, halving the elastic modulus of concrete and adhesive increases the prestress transfer length by 15.32% and 29.36%, respectively, while doubling these moduli decreases the transfer length by 8.62% and 18.45%, respectively. According to Equation (3), the effective bond stiffness *k*_e_ increases with the shear strength *k*_a_ of the adhesive layer and the shear strength *k*_c_ of the concrete cove, which leads to an increase in the stiffness coefficient Band, consequently, a reduction in the prestress transfer length. A shorter transfer length is associated with a more abrupt strain gradient at the CFRP end and a higher local shear stress, thereby increasing the risk of localized failure. As indicated by Equation (19), a higher CFRP elastic modulus yields a longer prestress transfer length: halving the modulus reduces the transfer length by 29.29%, whereas doubling it increases the transfer length by 41.43%. For a given level of applied prestress, a higher CFRP elastic modulus results in lower strain. When the CFRP modulus is halved, the shear stress at 20 mm from the CFRP end increases by 9.44%; conversely, doubling the modulus decreases the shear stress by 15.04%.

(2) CFRP cross-section and groove dimensions

**Figure 18:**
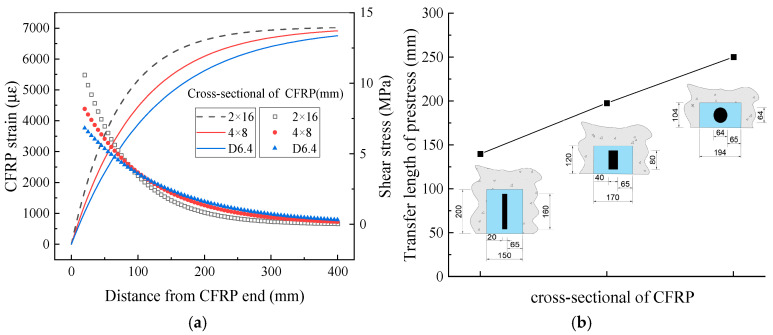
Effect of CFRP cross-section on prestress transfer behavior. (**a**) CFRP strain; (**b**) transfer length of prestress.

In NSM strengthening applications, the cross-sectional geometry of the strengthening material—typically rectangular, square, or circular (reinforcing bar)—significantly affects bond performance. Rectangular CFRP plates, which possess a higher ratio of bonding area to cross-sectional area compared to other shapes, exhibit superior bond characteristics. In addition to the 2 mm × 16 mm (width × height) rectangular plate used in this study, comparative analyses were performed using 4 mm × 8 mm rectangular plates and 6.4 mm diameter reinforcing bars. All three cross-sectional types were designed to have equal cross-sectional areas and adhesive layer thicknesses. Their respective bonding area-to-cross-sectional area ratios are 1.125, 0.75, and 0.625, with corresponding height-to-width ratios of 8, 2, and 1. As shown in Figure 18, the prestress transfer length increases with the width of the CFRP cross-section, exhibiting an approximately linear relationship with the bonding area-to-cross-sectional area ratio.

**Figure 19:**
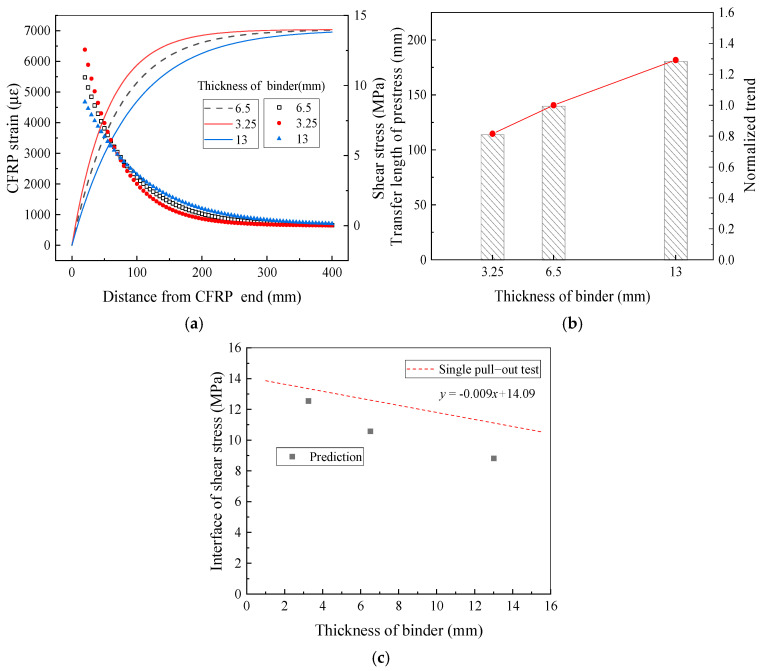
Effect of adhesive thickness on prestress transfer behavior. (**a**) CFRP strain; (**b**) transfer length of prestress; (**c**) comparison of shear stress.

Figure 19a,b demonstrate that increasing the adhesive layer thickness reduces its shear stiffness, resulting in a roughly linear increase in prestress transfer length. However, a thicker adhesive layer also enhances the deformation capacity and reduces the shear stress at the CFRP end. As previously reported [33,34] and illustrated in Figure 19c, the interface bearing capacity decreases with increasing adhesive layer thickness. Specimens with thinner adhesive layers exhibit peak shear stresses closer to the experimentally measured ultimate shear stress. Nevertheless, the use of excessively thin adhesive layers is not recommended due to the increased risk of damage and potential groove dimension errors.

(3) Degree of adhesive curing

Experimental results on the temperature-dependent performance of epoxy resin indicate that the time-dependent variation in the elastic modulus *E*(*t*) an be described by Equation (21), where room temperature is defined as 20 °C and time is measured in hours:

(21) $$E(t)=A_{1}-\frac{A_{2}}{{\left(1+\frac{x}{\alpha_{2}}\right)}^{\alpha_{1}}}=3-\frac{0.9607}{{\left(1+\frac{x}{36.07}\right)}^{5.4732}},$$

where α_1_ and α_2_ represent the time and size parameters, respectively, while *A*_1_ and *A*_2_ are fitting coefficients derived through linear regression analysis.

The effective bond stiffness *k*_e_, as defined by Equation (22), is derived from the shear stiffness ka of the adhesive:

(22) $$k_{a}(t)=\frac{G_{a}\cdot E(t)}{t_{a}}\to k_{e}(t)=\left[\frac{k_{c}}{1+\frac{k_{c}}{k_{a}(t)}}\right].$$

Accordingly, during the curing process of the epoxy resin, the prestress transfer length can be calculated using Equation (23):

(23) $$TL(t)=\frac{2}{B}\to B(t)=\sqrt{\frac{2k_{e}(t)}{a_{f}\cdot E_{f}}}.$$

**Figure 20:**
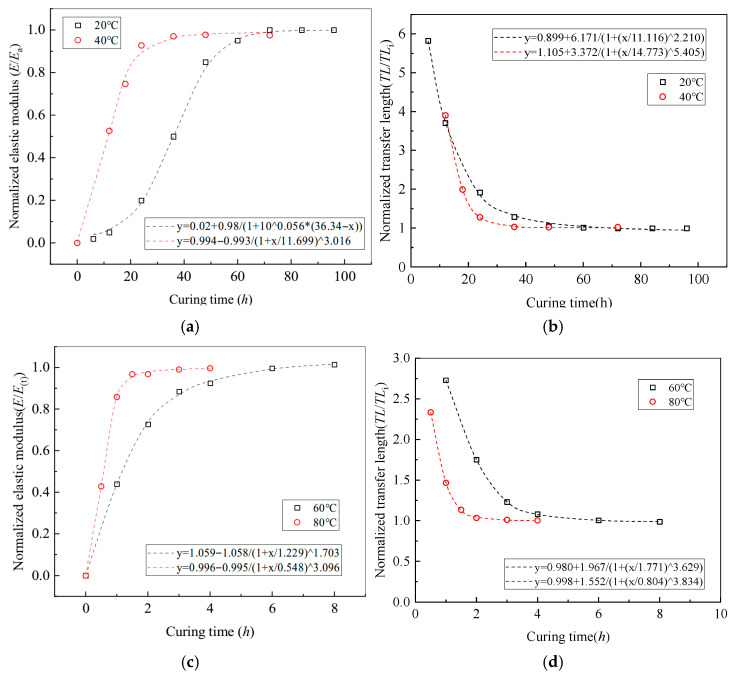
Effect of curing process on prestress transfer behavior. (**a**) Elastic modulus at 20 °C and 40 °C; (**b**) transfer length of prestress at 20 °C and 40 °C; (**c**) elastic modulus at 60 °C and 80 °C; (**d**) transfer length of prestress at 60 °C and 80 °C.

Figure 20 presents a normalized comparison of the elastic modulus of the epoxy resin and the prestress transfer length as functions of curing time at a constant temperature of 20 °C. As curing progresses, the degree of adhesive curing increases, resulting in a corresponding rise in effective bond stiffness and a gradual reduction in prestress transfer length. These trends are well characterized by exponential fitting models, as expressed in Equations (24) and (25):

(24) $$y=0.02+\frac{0.98}{1+10^{0.056\cdot (36.34-x)}},$$

(25) $$y=1+4.82\cdot e^{-(\frac{x-6}{10.93})}.$$

It should be emphasized that this analysis considers only the short-term mechanical response of NSM-CFRP systems under monotonic elevated temperature. The assumptions of linear elasticity, perfect bond, and no-slip conditions do not account for (i) nonlinear bond–slip or viscoelastic creep/relaxation of the epoxy, (ii) cyclic thermal loading, (iii) moisture ingress, or (iv) freeze–thaw action. Such coupled environmental effects can progressively degrade interfacial stiffness, increase the prestress transfer length, and alter failure modes. Future research should therefore incorporate nonlinear bond–slip laws or viscoelastic adhesive models within finite-element simulations and conduct long-term tests under combined temperature–humidity cycles and freeze–thaw regimes to establish durability-based design criteria.

## 5. Conclusions

Comprehensive experimental investigations and theoretical analyses were conducted to quantify the effects of curing and service temperatures on epoxy performance. Utilizing a calibrated stiffness model, the cascading impacts on CFRP strain distribution and prestress transfer length were systematically elucidated. The principal conclusions are as follows:

(1) The curing time required for epoxy resin to achieve full strength exhibits an inverse exponential relationship with curing temperature. While epoxy performance remains relatively stable across a range of curing temperatures, only a minor reduction (less than 10%) is observed when the temperature exceeds 80 °C. In contrast, service temperature exerts a significant influence: both tensile and shear strengths of the epoxy decrease markedly as ambient temperature increases, with negligible tensile capacity observed above 80 °C.
(2) Introducing the concept of effective bond stiffness enables accurate characterization of adhesive–concrete bonding performance and facilitates precise simulation of shear stress distribution at the CFRP–concrete interface under prestress. The proposed prestress reduction coefficient further extends the model’s applicability, enabling analysis of structures with varying prestress levels and gradually anchored prestressed strengthening systems.
(3) Parametric analyses demonstrate that the elastic modulus of CFRP exerts the greatest influence on shear stress distribution, with prestress transfer length increasing proportionally with the modulus. For a constant strengthening ratio, CFRP sections with larger bonding areas provide superior interfacial performance, whereas increasing adhesive layer thickness reduces effective bond stiffness and diminishes the overall performance of the bonding interface.
(4) The prestress transfer behavior under varying curing temperatures and durations is governed by changes in the adhesive’s elastic modulus. As curing temperature increases and curing time extends, the effective bond stiffness of the adhesive increases accordingly, while the degree of prestress transfer decreases, both following an exponential trend.
(5) Based on the validated model and experimental data, the following concise design recommendations are proposed: limit service temperature to ≤60 °C (short-term exposure up to 80 °C is permissible only if prestress is reduced or a higher-*T*_g_ adhesive is employed); provide an adhesive layer thickness of at least 2 mm; ensure full cure (≥96 h at 20 °C or 22 h at 40 °C); limit prestress to 50% of the CFRP ultimate strength; and provide mechanical anchorage if higher prestress levels are required. Wide, thin rectangular NSM strips are recommended when the cross-sectional area is fixed.

## Figures and Tables

**Table 1:** Material properties.

Materials	Tensile Strength/MPa	Compressive Strength/MPa	Elongation/%	Elastic Modulus/GPa
CFRP strip	2561.1	-	1.96	142.2
Epoxy resin	31.9	-	1.47	11.2
C40 concrete		41.9	-	33.5
HRB400 steel bar	540	442	16	201.9

## Data Availability

The original contributions presented in this study are included in the article. Further inquiries can be directed to the corresponding author.
